# Influenza HA Subtypes Demonstrate Divergent Phenotypes for Cleavage Activation and pH of Fusion: Implications for Host Range and Adaptation

**DOI:** 10.1371/journal.ppat.1003151

**Published:** 2013-02-14

**Authors:** Summer E. Galloway, Mark L. Reed, Charles J. Russell, David A. Steinhauer

**Affiliations:** 1 Department of Microbiology and Immunology, Emory University School of Medicine, Atlanta, Georgia, United States of America; 2 Department of Infectious Diseases, St. Jude Children's Research Hospital, Memphis, Tennessee, United States of America; 3 Department of Microbiology, Immunology, and Biochemistry, University of Tennessee Health Science Center, Memphis, Tennessee, United States of America; University of Wisconsin-Madison, United States of America

## Abstract

The influenza A virus (IAV) HA protein must be activated by host cells proteases in order to prime the molecule for fusion. Consequently, the availability of activating proteases and the susceptibility of HA to protease activity represents key factors in facilitating virus infection. As such, understanding the intricacies of HA cleavage by various proteases is necessary to derive insights into the emergence of pandemic viruses. To examine these properties, we generated a panel of HAs that are representative of the 16 HA subtypes that circulate in aquatic birds, as well as HAs representative of the subtypes that have infected the human population over the last century. We examined the susceptibility of the panel of HA proteins to trypsin, as well as human airway trypsin-like protease (HAT) and transmembrane protease, serine 2 (TMPRSS2). Additionally, we examined the pH at which these HAs mediated membrane fusion, as this property is related to the stability of the HA molecule and influences the capacity of influenza viruses to remain infectious in natural environments. Our results show that cleavage efficiency can vary significantly for individual HAs, depending on the protease, and that some HA subtypes display stringent selectivity for specific proteases as activators of fusion function. Additionally, we found that the pH of fusion varies by 0.7 pH units among the subtypes, and notably, we observed that the pH of fusion for most HAs from human isolates was lower than that observed from avian isolates of the same subtype. Overall, these data provide the first broad-spectrum analysis of cleavage-activation and membrane fusion characteristics for all of the IAV HA subtypes, and also show that there are substantial differences between the subtypes that may influence transmission among hosts and establishment in new species.

## Author Summary

IAV is associated with significant morbidity and mortality, and represents a challenging public health threat that affects social and economic welfare each year, particularly during IAV pandemics. Although we know that all human strains derive, either directly or via intermediate hosts, from avian viral sources, we know very little about the phenotypic characteristics of the 16 HA subtypes that circulate in aquatic birds and have potential to infect mammals. HA membrane fusion properties, in conjunction with the characteristics for protease activation of HA, a requirement for fusion, are critical factors involved in the ecology and transmission of IAVs, and need to be understood if we are to derive explanations for how pandemic viruses emerge in humans. We examined the cleavage-activation and membrane fusion characteristics for the 16 HA subtypes by transiently expressing HA proteins in cells. Our findings show that the cleavability of the HAs vary considerably between subtypes and depending on the protease. Additionally, analysis of the pH of fusion for each subtype showed that HA stability varied significantly among the subtypes, as well as within subtypes from viruses isolated from different species. Overall, these data have implications for host range, potential for adaptation, and persistence in natural environments.

## Introduction

Influenza A virus (IAV) is a significant human pathogen that is maintained in nature via an enzootic replication cycle among aquatic birds [1]. The full complement of IAV surface glycoproteins, the hemagglutinin (HA) and neuraminidase (NA), is represented in wild aquatic birds, of which there are currently 16 HA and nine NA subtypes. The recent identification of IAV genetic material from bats in Guatemala suggests the circulation of additional antigenically distinct HA and NA subtypes [2]. Although aquatic birds are believed to be the natural reservoir for IAV, sporadic cross-species transmission events have led to the spread of IAVs to other avian species as well as mammals [1]. These cross-species transmission events are often characterized by the rapid evolution of viral proteins for adaptation to the new host, which may be influenced by a variety of selective pressures, including differences in availability and structure of host cell receptors, variations in host cell transcription/translation factors, variations in host cell entry mechanisms, and sites of replication. To date, through either direct transmission or via an intermediate host, only the H1, H2, and H3 HA subtypes and the N1 and N2 subtypes are known to have become established in the human population. Although H5 and H7 subtypes have infected humans, often with considerable morbidity and mortality, they have yet to show efficient transmission between humans. Recent studies have shown that recombinant viruses containing the H5 HA from A/VietNam/1203/2004 (H5N1) or A/Indonesia/5/2005 (H5N1) were capable of transmitting more efficiently via respiratory droplet between ferrets if the HA contained mutations that confer Siaα2,6Gal binding (N224K and Q226L for H5^VN^ or Q222L and G224S for H5^IN^), loss of a glycosylation site within the head domain (N158D for H5^VN^ or T156A or N154K for H5^IN^), and a mutation that increased the stability of the H5 HA (T318I for H5^VN^) [3], [4].

**Figure 1:**
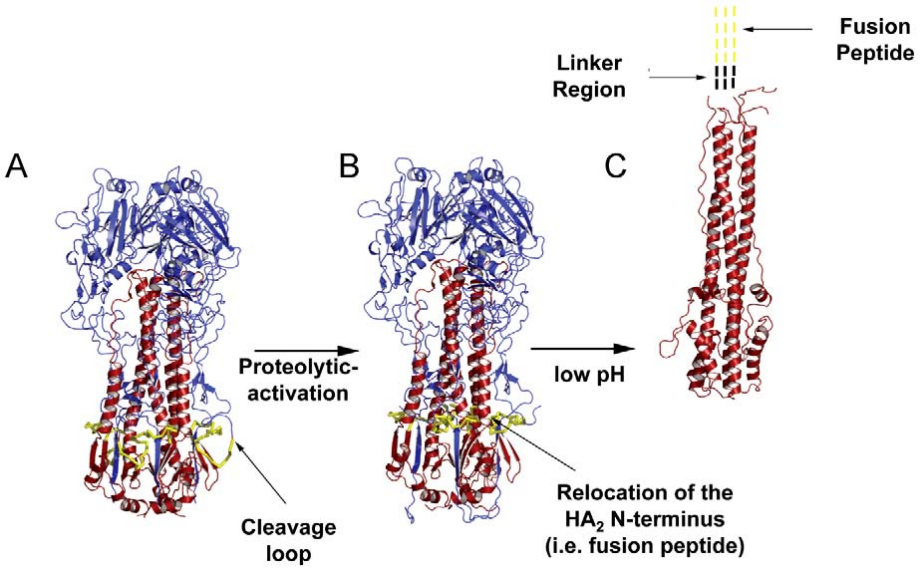
Structures of the conformations assumed by HA during the virus life cycle. Trimeric HA molecule is shown as a ribbon diagram, with the HA_1_ subunit colored in blue, the HA_2_ subunit colored in red, and residues corresponding to the fusion peptide colored in yellow. (**A**) The site within the trimeric HA molecule that acts as a substrate for activating protease (i.e. the cleavage loop) is denoted with an arrow. (**B**) Upon proteolytic activation within the cleavage loop, the N-terminus of the newly generated HA_2_ subunit is relocated to the trimer interior where it forms extensive contacts with a network of residues within the fusion peptide pocket. This forms the metastable conformation that is primed for fusion. (**C**) Upon exposure to low pH, the HA undergoes a series of conformational changes that result in the extrusion of the fusion peptide for mediating membrane fusion between the virion and endosomal membranes.

While several viral proteins have been shown to acquire adaptive mutations that mediate more efficient IAV replication in specific hosts, the best characterized of these is the HA protein [5]–[13]. The IAV HA protein is responsible for mediating two primary events during virus entry: 1) binding to cell surface glycan receptors containing terminal sialic acid and 2) mediating membrane fusion between the viral and endosomal membranes to release the ribonucleoprotein core of the virion into the cell. The IAV HA protein is perhaps the most extensively characterized class I viral fusion protein [14]–[17]. As such, high-resolution structures of three distinct conformations of the HA during the replication cycle have been solved: uncleaved HA_0_, pre-fusion HA_1_+HA_2_, and post-fusion HA_2_ (Figure 1) [18]–[21]. Like all class I fusion proteins, IAV HA proteins associate in the endoplasmic reticulum during biogenesis where they non-covalently oligomerize as homotrimers. The HA protein is synthesized in infected cells as a single polyprotein precursor, HA_0_. Cleavage of each HA_0_ monomer into the disulfide-linked HA_1_ and HA_2_ subunits is required to activate the membrane fusion potential of the HA, and therefore virus infectivity [22]–[24]. All HA proteins are cleaved by activating proteases within a membrane-proximal surface loop at an R (or K) residue located adjacent to the G residue that constitutes the N-terminus of the newly generated and highly conserved fusion peptide of the HA_2_ subunit (Figure 1A). For low-pathogenic IAV strains, the cleavage site consists of a monobasic R/K residue, which may be cleaved by trypsin-like serine proteases for activation. Generally, these HAs are transported to the plasma membrane in their uncleaved, HA_0_ form [25], [26]; however, recent data regarding cleavage of HA_0_ by the type II transmembrane serine protease, TMPRSS2, indicates that TMPRSS2 cleaves HA_0_ in the secretory pathway within the cell [27]. For highly pathogenic IAV strains, the cleavage site consists of a polybasic stretch of amino acids that may be cleaved by ubiquitous intracellular furin-like serine proteases, such as furin and PC6 [28]–[31]. These HA proteins are cleaved intracellularly within the trans-Golgi network. Cleavage of HA_0_ by ubiquitous proteases increases the likelihood for systemic infection, but HA proteins requiring cleavage by trypsin-like proteases are generally restricted to specific sites where these enzymes are expressed. In addition to the amino acid composition of the cleavage site, the size of the cleavage loop and the presence or absence of nearby carbohydrates can alter the accessibility of activating protease [24], [32]–[35].

Upon cleavage of the trimeric HA_0_ molecule, the newly generated hydrophobic HA_2_ N-terminal domain (i.e. the fusion peptide) is relocated to a cavity in the trimer interior where it forms extensive contacts with residues in the metastable conformation, which renders the molecule fusogenic (Figure 1B) [18], [21]. Cleaved HA molecules are able to enter host cells via the endocytic pathway, whereby the HA protein binds to cell surface receptors containing terminal sialic acid moieties and, following internalization, undergoes irreversible conformational changes in response to acidification of the endosomal vesicle, thus triggering fusion of the viral and endosomal membranes (Figure 1C). Consequently, cleavage-activation and membrane fusion are intimately associated with one another, as uncleaved HA molecules are unable to undergo the necessary acid-induced conformational changes required for membrane fusion and virus infectivity [36].

There are a number of candidate proteases potentially involved in cleavage-activation of the IAV HA_0_ protein, and the use of any particular protease is likely dependent on the route of infection and the site of replication. Notable examples include human airway trypsin-like protease (HAT), which is expressed on ciliated epithelial cells [37], transmembrane protease, serine 2 (TMPRSS2), which is expressed on epithelial cells of the respiratory, gastrointestinal, and urogenital tracts [37], tryptase Clara, which is expressed in Clara cells of the bronchiolar epithelium [38], as well as the ubiquitously-expressed furin protease, which displays specificity for HA proteins containing polybasic cleavage sites. In addition, cleavage of IAV HA_0_ in embryonated chicken eggs has been shown to occur by a Factor Xa-like protease [39], [40]. More recently, it was shown that the secreted, catalytic domain of matriptase, a member of the type II transmembrane serine protease family, was capable of both direct cleavage of the influenza HA as well as indirect cleavage by way of promoting the activation of thrombolytic zymogens that have been shown to cleave HA [41]. Despite what is known, it has been difficult to unequivocally specify the identity of activating proteases in natural hosts, and the extent to which HAs of different subtypes and strains are cleaved by trypsin-like proteases has not been examined rigorously.

In addition to the availability and cleavage efficiency of activating proteases, we also know that different HA subtypes display a range of stability phenotypes, which are related to pH and temperature, and therefore have varying fusion characteristics and kinetics of inactivation [42], [43]. Together, these properties may reflect the broad ecological and host environments in which these viruses persist and replicate, as well as affect the ability, or at least be a contributing factor in the ability of new viruses to transmit or adapt to new species. As such, understanding the susceptibility of HAs from different subtypes to proteases found in natural sites of infection is necessary to derive insight into mechanisms of pathogenicity, ease of cross-species transmissibility, and relative fusogenic capabilities within distinct sites of virus replication.

The pH of membrane fusion as well as the requirement of protease activation, although well established, has only been examined for a limited number of subtypes. Therefore, to examine the subtypes in greater detail, as well as to discern potential differences between avian-origin and mammalian-origin HAs, we have generated a panel of wild-type HA clones, along with their cognate NA, that are representative of the 16 HA subtypes that circulate in avian species, as well as several from human isolates of the H1, H2, H3, and H5 subtypes. In this study, we examined the efficiency of cleavage-activation by a variety of proteases as well as the pH at which membrane fusion occurs.

## Results

To examine the cleavage-activation and membrane fusion properties of HA proteins of each subtype, we cloned the HA and NA genes from influenza A viruses of avian-origin that were representative of each subtype. As these properties have not been examined systematically for all avian HA subtypes in a single study, we chose to use the WHO reference strains for each subtype for these initial characterizations. We also examined the HAs from several influenza A viruses of human-origin representing each of the three HA subtypes that have caused pandemics and circulated in humans over the past century, as well as an H5N1 human isolate (Table 1). HA and NA genes were cloned into the pCAGGS expression vector, as described in Materials and Methods.

**Table 1:** Origin of HA and NA proteins examined and HA cleavage site sequence.

Subtype	HA and NA donor	HA Cleavage site sequence^a^							
		P4	P3	P2	P1	P1′	P2′	P3′	P4′
H1N1	A/duck/Alberta/35/1976	I	Q	S	**R**	G	L	F	G
H2N3	A/duck/Germany/1215/1973	I	E	S	**R**	G	L	F	G
H3N8	A/duck/Ukraine/1/1963	K	Q	T	**R**	G	L	F	G
H4N6	A/duck/Czechoslovakia/1/1956	K	A	S	**R**	G	L	F	G
H5N3	A/tern/South Africa/1961	R	Q	K	**R**	G	L	F	G
H6N5	A/shearwater/Australia/1/1972	I	E	T	**R**	G	L	F	G
H7N3	A/turkey/England/1963	R	R	R	**R**	G	L	F	G
H8N4	A/turkey/Ontario/6118/1968	V	E	P	**R**	G	L	F	G
H9N2	A/turkey/Wisconsin/1/1966	V	S	S	**R**	G	L	F	G
H10N7	A/chicken/Germany/N/1949	V	Q	G	**R**	G	L	F	G
H11N6	A/duck/England/1/1956	I	A	S	**R**	G	L	F	G
H12N5	A/mallard/Sweden/81/2002	A	Q	D	**R**	G	L	F	G
H13N6	A/gull/Maryland/704/1977	I	S	N	**R**	G	L	F	G
H14N5	A/duck/Astrakhan/263/1982	K	Q	A	**K**	G	L	F	G
H15N8	A/duck/Australia/341/1983	I	H	T	**R**	G	L	F	G
H16N3	A/black-headed gull/Sweden/5/1999	V	G	E	**R**	G	L	F	G
H1N1	A/Puerto Rico/8/1934	I	Q	S	**R**	G	L	F	G
H1N1	A/WSN/1933	I	Q	Y	**R**	G	L	F	G
H1N1	A/California/04/2009	I	Q	S	**R**	G	L	F	G
H1N1	A/Georgia/F32551/2012	I	Q	S	**R**	G	L	F	G
H1N1	A/Pennsylvania/08/2008	I	Q	S	**R**	G	L	F	G
H2N2	A/Japan/305/1957	I	E	S	**R**	G	L	F	G
H3N2	A/Aichi/2/1968	K	Q	T	**R**	G	L	F	G
H3N2	A/Victoria/3/1975	K	Q	T	**R**	G	I	F	G
H5N1	A/VietNam/1204/2004	R	K	K	**R**	G	L	F	G

a General nomenclature of substrate cleavage site, with cleavage occurring between the P1 and P1′ positions. The P1 positions are shown in bold type.

### Trypsin-mediated cleavage of HA subtypes

Although the requirement for cleavage-activation of HA to prime the molecule for infectivity is well documented [22], [23], [44]–[46], the extent to which HA is cleaved by trypsin, as well as by more biologically relevant proteases has not been extensively examined for all subtypes and is likely to influence the fusogenicity of HA proteins.

**Figure 2:**
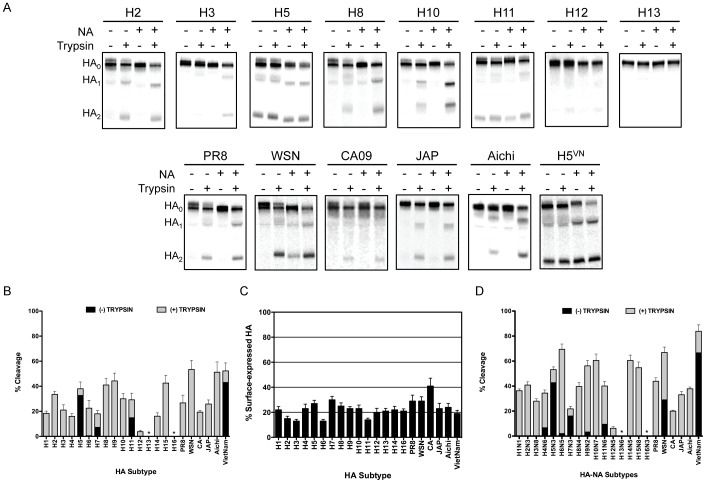
Analysis of trypsin-mediated cleavage-activation of HA in the absence and presence of NA. (**A**) BHK cells were transfected with 1.0 µg HA plasmid or co-transfected with 1.0 µg HA plasmid and 0.1 µg NA plasmid. At 16 hours post transfection, cells were starved for methionine and cysteine, and were pulse labeled with 25 µCi [^35^S]-methionine for 15 min, followed by a 3 hr chase period. Where indicated, radiolabeled, HA-expressing cell monolayers were treated with 5 µg/ml TPCK-Trypsin for 15 min. Total cellular HA protein was immunoprecipitated with a HA-specific antibody and resolved on 12% SDS-PAGs. The gels were fixed and dried, and radiolabeled proteins were visualized by phosphorimaging. Representative images of gels are shown with the HA subtype denoted at the top of each gel and the identity of each band is denoted on the left. (**B**) Quantitation of trypsin-mediated cleavage of HA in the absence of NA. Quantitative analysis was performed on three independent experiments. For each HA, the intensity of the bands corresponding to HA_0_ and HA_2_ were normalized to their respective methionine content and the percent cleavage (mean ± standard deviation) is expressed as a percentage of the total cellular HA, by the equation (HA_2_/(HA_2_+HA_0_))×100%. * denotes no detectable cleavage. (**C**) Quantitation of surface-expressed HA. BHK cells were transfected with 1.0 µg HA plasmid. At 16 hours post transfection, cells were starved for methionine and cysteine, and were pulse labeled with 25 µCi [^35^S]-methionine for 15 min, followed by a 3 hr chase period. Cell surface proteins were then biotinylated and total HA protein was immunoprecipitated with a HA-specific antibody, followed by immunoprecipitation with streptavidin, and resolved on 12% SDS-PAGs. The gels were fixed and dried, and radiolabeled proteins were visualized by phosphorimaging. Quantitative analysis of three independent experiments is shown. The percentage of surface-expressed HA was determined by dividing the amount of surface-expressed HA by the total HA. (**D**) Quantitation of trypsin-mediated cleavage of HA in the presence of NA. Quantitative analysis was performed as described in (B). * denotes no detectable cleavage.

To examine the susceptibility of our panel of HAs to trypsin, BHK cells were transfected with an HA-expressing plasmid and then transfected cell proteins were metabolically labeled with [^35^S]-Met, followed by a chase period, and either left untreated or treated with TPCK-trypsin. HA protein was then immunoprecipitated from cell lysates and resolved by SDS-PAGE. Figure 2A shows the trypsin cleavage profiles for a representative set of the HAs examined. Quantitative analysis of three independent experiments showed that the efficiency of HA cleavage varied substantially from subtype to subtype, ranging from 0–53% of total cellular HA (Figure 2B). To assess whether the differences observed in cleavage efficiency were due to differences in the level of total HA expressed on the surface of BHK cells, we examined the percent of each HA that was expressed on the surface of transfected cells, as described in Material and Methods. As shown in Figure 2C, the levels of HA expressed on the surface of transfected cells did not vary considerably, and in cases of significant variability, there was no positive correlation between cleavage efficiency and surface expression.

**Figure 3:**
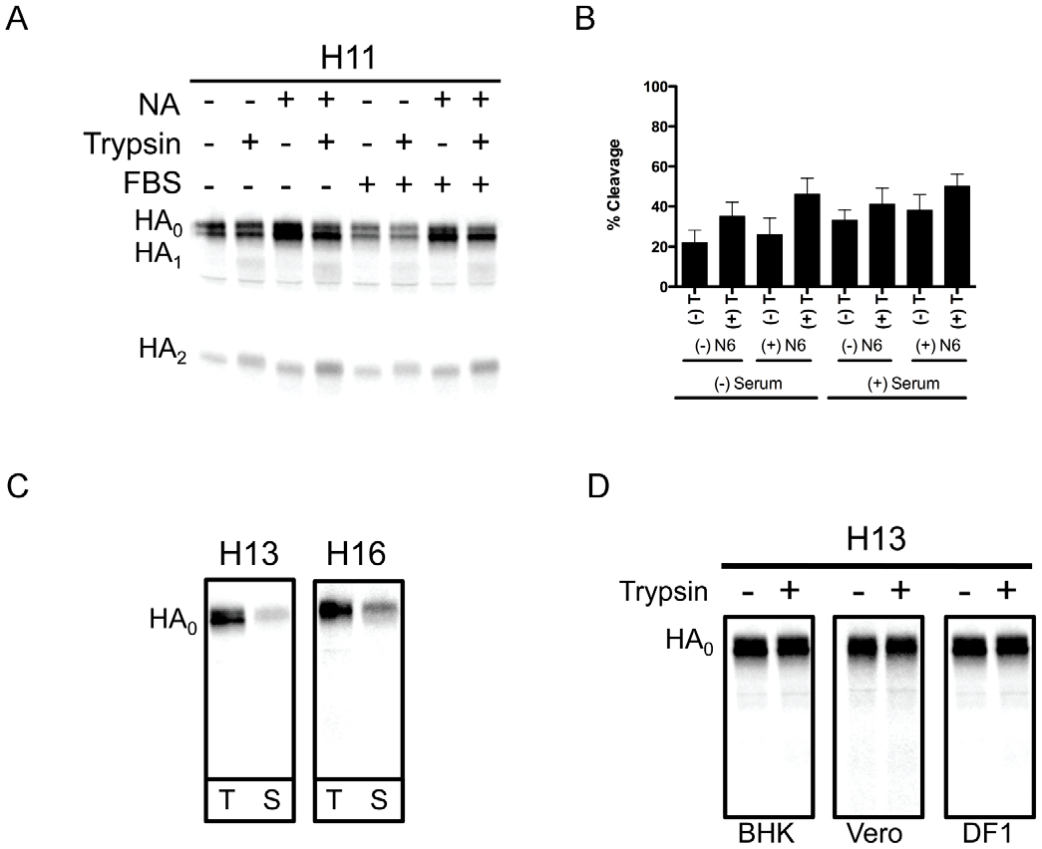
Analysis of proteolytic cleavage of H11 HA and surface expression of H13 and H16 HAs. (**A**) BHK cells were transfected with 1.0 µg H11 HA plasmid or co-transfected with 1.0 µg H11 HA plasmid and 0.1 µg N6 NA plasmid. Experimental details are identical to those stated in Figure 2A, except that during the chase period media either lacking serum or containing 10% fetal bovine serum was used. The conditions assayed for the samples in each lane are denoted at the top of the gel and the identity of each band is denoted on the left. (**B**) Quantitation of H11 HA cleavage in the presence and absence of NA, the presence and absence of 10% fetal bovine serum, and in the presence and absence of trypsin. Quantitative analysis was performed on three independent experiments. For each HA, the intensity of the bands corresponding to HA_0_ and HA_2_ were normalized to their respective methionine content and the percent cleavage (mean ± standard deviation) is expressed as a percentage of the total cellular HA, by the equation (HA_2_/(HA_2_+HA_0_))×100%. (**C**) BHK cells were transfected with 1.0 µg H13 or H16 HA plasmid. At 16 hours post transfection, cells were starved for methionine and cysteine, and were pulse labeled with 25 µCi [^35^S]-methionine for 15 min, followed by a 3 hr chase period. Cell surface proteins were then biotinylated and total HA protein was immunoprecipitated with a HA-specific antibody, followed by immunoprecipitation with streptavidin, and resolved on 12% SDS-PAGs. The gels were fixed and dried, and radiolabeled proteins were visualized by phosphorimaging. The HA subtype is denoted at the top of each gel and the identity of each band is denoted on the left. T = Total HA, S = Surface expressed HA. (**D**) BHK, Vero, or DF-1 cells were transfected with 1.0 µg of H13 HA plasmid. Experimental details are the same as described in 2A.

As expected, we observed that the H5 and H7 HAs were cleaved in the absence of trypsin due to the presence of polybasic cleavage sites. However, further cleavage was detected following exposure to trypsin, suggesting that there was a percentage of H5 and H7 HAs that were expressed on the cell surface in an uncleaved form (Figure 2A and 2B). Surprisingly, we also observed HA cleavage for the H11 HA in the absence of trypsin, despite the fact that this HA does not contain a polybasic cleavage site (Table 1). For these experiments, serum-containing media was used during the chase period, which provides for the possibility that the H11 HA is being cleaved by a protease present in serum, such as plasminogen/plasmin. Therefore, to investigate this possibility, we compared the HA cleavage profile for H11 using media lacking or containing serum during the chase period. As shown in Figures 3A and 3B, the cleavage profiles for the H11 HA in the absence of serum are indistinguishable from those in the presence of serum, regardless of the presence of NA. These data indicate that the H11 HA is being cleaved by a protease other than those found in serum, or that a protease present in serum is sequestered by a protein on the surface of H11 HA-transfected BHK cells that is not removed by our washes prior to the starvation period.

Another interesting observation involved the H13 and H16 HAs, for which no trypsin-mediated cleavage was detected (Figure 2A and 2B). Data shown in Figure 2C confirm that these HAs were expressed on the surface of BHK cells and therefore accessible to exogenous trypsin. In addition to the data shown in Figure 2C, images of the gels of immunoprecipitated total and surface-expressed H13 and H16 are shown in Figure 3C. We also examined whether trypsin-mediated cleavage of the H13 and H16 HAs was affected by the cell type in which HA was synthesized. In addition to BHK cells, we examined cleavage in both Vero and DF-1 cells using the same method described above. Figure 3D shows the data for the H13 HA, which indicates that the cell type in which HA was synthesized had no detectable effect on its ability to be cleaved by exogenous trypsin. Expression of H13 and H16 on the surface of Vero and DF-1 cells was also confirmed (data not shown). The observed lack of trypsin-mediated cleavage of the H13 and H16 HAs was not specific to those included in the initial characterization, as we also examined trypsin-mediated cleavage of two additional H13 HAs and one additional H16 HA, none of which displayed any detectable cleavage (data not shown).

### Effects of NA on trypsin-mediated cleavage of HA

It has been shown that co-expression of NA may influence the efficiency of HA cleavage [47]. Therefore, we also examined trypsin-mediated cleavage of HA in the presence of its cognate NA. Experiments were conducted as described above, except that a plasmid expressing NA was co-transfected with the HA-expressing plasmid at a 1∶10 ratio. Figure 2A shows the trypsin cleavage profiles of HA when co-expressed with its cognate NA. Quantitative analysis of three independent experiments showed that, for the majority of HAs examined, co-expression of HA and NA led to an increase in the percent of total cellular HA that was cleaved by trypsin (Figure 2D). As expected, co-expression of WSN HA and NA resulted in HA cleavage in the absence of trypsin due to the plasminogen binding activity associated with the WSN NA [47]; however, HA cleavage was significantly enhanced by the addition of trypsin when co-expressed with WSN NA. Notable exceptions to the NA-enhanced cleavage phenotype were the H13 and H16 HAs, for which co-expression of NA did not facilitate trypsin-mediated cleavage of HA (Figure 2A and 2D). We also examined cleavage of the H13 and H16 HAs in the presence of the WSN NA to determine whether its plasminogen/plasmin binding activity could facilitate cleavage of these HAs, and similar to the data with their cognate NAs, no cleavage was observed (data not shown).

### Effects of NA on cell surface expression of HA

**Figure 4:**
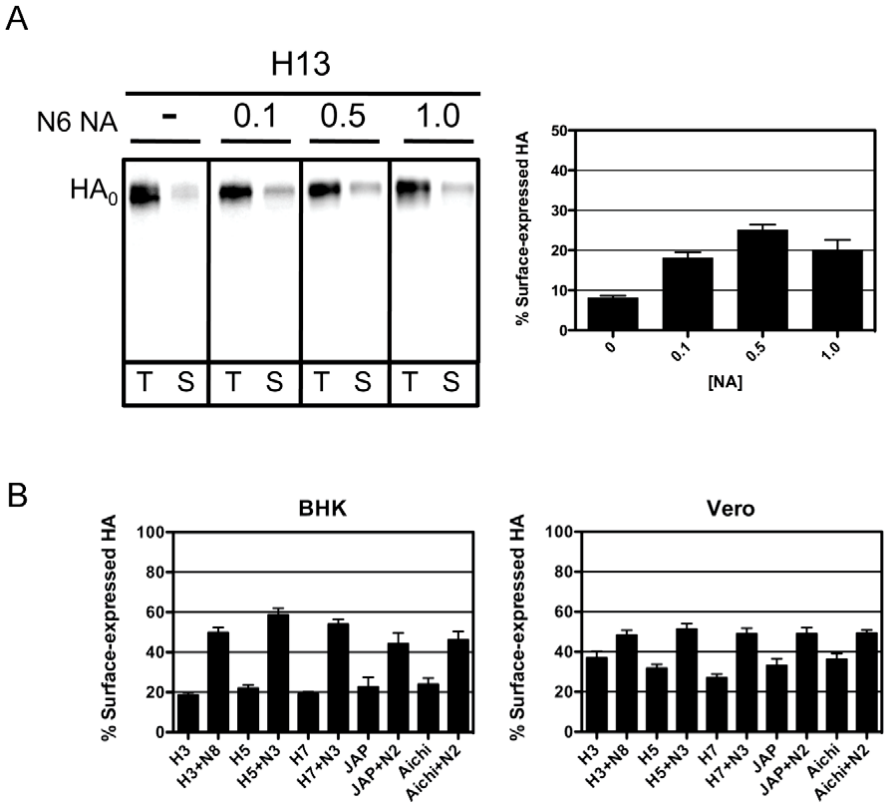
Effect of co-expressing NA on cell surface expression of HA. (**A**) BHK cells were transfected with 1.0 µg H13 plasmid only, or co-transfected with 0.1 µg, 0.5 µg, or 1.0 µg N6 plasmid. At 16 hours post transfection, cells were starved for methionine and cysteine, and were pulse labeled with 25 µCi [^35^S]-methionine for 15 min, followed by a 3 hr chase period. Cell surface proteins were then biotinylated and total HA protein was immunoprecipitated with a HA-specific antibody, followed by immunoprecipitation with streptavidin, and resolved on 12% SDS-PAGs. The gels were fixed and dried, and radiolabeled proteins were visualized by phosphorimaging. The amount of NA plasmid is denoted at the top of each gel and the migration of HA_0_ is denoted to the left of the gel. T = Total HA, S = Surface expressed HA. The graph to the right of the gel shows the quantitative analysis of three independent experiments. The percentage of surface-expressed HA was determined by dividing the amount of surface-expressed HA by the total HA. (**B**) Experimental details are the same as in A, except that BHK or Vero cells were transfected with 1.0 µg H3, H5, H7, Japan, or Aichi HA plasmid only or in combination with 0.1 µg of cognate NA plasmid. The data shown represents the quantitative analysis of three independent experiments. Quantitation was performed exactly as stated in (A).

As mentioned, an increase in the percent cleavage of HA was observed for nearly all HAs examined when co-expressed with its cognate NA compared to when HA was expressed alone. This was not altogether surprising given that the presence of NA has been shown to affect cleavage of HA [47], [48]. However, during our studies to asses cleavage and surface expression of the H13 and H16 HAs, we observed that when the HA and NA proteins were co-expressed, the level of HA expressed on the cell surface was greater than when HA was expressed alone, providing a potential explanation for the increase in cleavage observed in the presence of NA (i.e. more HA on the surface and available to exogenous trypsin). To investigate this phenotype further, we co-expressed the H13 HA at varying ratios with its cognate NA (1∶0.1, 1∶0.5, and 1∶1), and found that co-expression of HA and NA, even at the lowest ratio tested, led to greater levels of HA expressed on the cell surface (Figure 4A). We also performed a similar experiment using the H3, H5, H7, Japan, and Aichi HA-NA pairs, in which we either transfected 1.0 µg HA plasmid alone or co-transfected 1.0 µg HA plasmid and 0.1 µg NA plasmid, and examined the level of HA expressed on the surface of BHK and Vero cells, as described in Materials and Methods. The data in Figure 4B show that co-expression of these HA and NA pairs also result in greater levels of cell surface-expressed HA.

### HA cleavage mediated by human serine proteases

**Figure 5:**
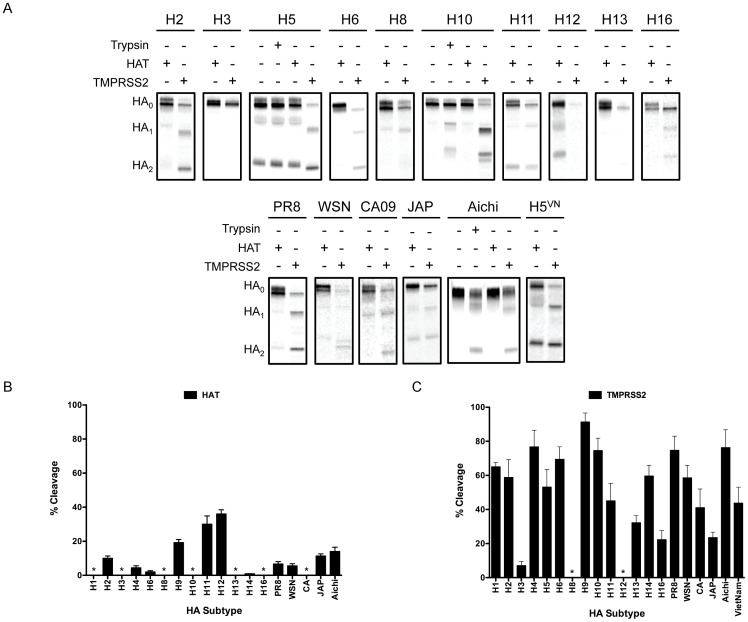
Analysis of HA cleavage by human serine proteases, HAT and TMPRSS2. (**A**) BHK cells were transfected with 1.0 µg HA plasmid and 0.25 µg of either HAT or TMPRSS2 expression plasmid. At 16 hours post transfection, cells were starved for methionine and cysteine, and were pulse labeled with 25 µCi [^35^S]-methionine for 15 min, followed by a 3 hr chase period. Total HA protein was immunoprecipitated with a HA-specific antibody and resolved on 12% SDS-PAGs. The gels were fixed and dried, and radiolabeled proteins were visualized by phosphorimaging. The identity of each HA is denoted above the gel and the migration of HA_0_, HA_1_, and HA_2_ is denoted to the left of the gel. (**B**) Quantitation of HAT-mediated cleavage of HA. Quantitative analysis was performed on three independent experiments. For each HA, the intensity of the bands corresponding to HA_0_ and HA_2_ were normalized to their methionine content and the percent cleavage (mean ± standard deviation) is expressed as a percentage of the total cellular HA, by the equation (HA_2_/(HA_2_+HA_0_))×100%. * denotes no detectable cleavage. (**C**) Quantitation of TMPRSS2-mediated cleavage of HA. Quantitative analysis was performed on three independent experiments, as described in (B). The percent cleavage shown for the H5 and H5^VN^ HAs was determined by subtracting the percent cleavage observed in the absence of trypsin from the percent cleavage observed in the presence of TMPRSS2.

In addition to examining HA cleavage with trypsin, we examined HA cleavage efficiency by human airway trypsin-like protease (HAT) and transmembrane serine protease 2 (TMPRSS2), which are both type II transmembrane serine proteases that have been experimentally shown to cleave IAV HA [37]. HAT and TMPRSS2 are both expressed in the mammalian airway epithelium, as well as in many other epithelial tissues throughout the body, making them viable candidates for the proteolytic activation of IAV HA *in vivo* [49]–[51]. To examine the susceptibility of our panel of HA subtypes to HAT and TMPRSS2, BHK cells were co-transfected with a HA-encoding plasmid and a plasmid expressing HAT or TMPRSS2, and transfected cell proteins were metabolically labeled and isolated as stated previously and in Materials and Methods. Figure 5A shows the HAT and TMPRSS2 cleavage profiles for a representative set of HA proteins examined, and indicate that the efficiency of HA cleavage and profiles of digestion products were dependent on the protease and varied substantially among subtypes. Figures 5B and 5C summarize the percent cleavage from quantitative analysis of three independent experiments. In general, HA proteins co-expressed with HAT were cleaved to a lesser extent than when co-expressed with TMPRSS2. The substantial disparity in cleavage efficiency by the two human proteases is most likely due to a combination of differences in HA cleavage site sequences as well as protease accessibility to HA. As mentioned previously, TMPRSS2 has proteolytic activity within the secretory pathway, while HAT is only active once on the cell surface; therefore, a higher percentage of HA, namely intracellular HA, is available for cleavage by TMPRSS2. The exception to the aforementioned trend was the H12 HA, which was the only HA examined to show greater cleavage by HAT than by TMPRSS2; the H12 HA was also poorly cleaved by trypsin (Figure 2A and B). Quantitation of TMPRSS2-mediated cleavage for the H12 HA was not possible, as co-expression resulted in an overall decrease in HA protein that was immunoprecipitated, and while quantitation of the TMPRSS2-cleaved WSN HA was possible, the overall amount of HA immunoprecipitated was significantly decreased (See H12 and WSN in Figure 5A). While we do not have a definitive explanation for the lower levels of total HA that were observed in the presence of TMPRSS2, it is possible that HA is being degraded by TMPRSS2 or that TMPRSS2 is cleaving at multiple sites within the HA, which may render the HA more prone to degradation within the cell.

Although the H13 and H16 HAs were not cleaved by trypsin or HAT, TMPRSS2 was capable of cleavage activation of both HAs at approximately 32% and 22% of total HA, respectively (Figure 5A–C). Interestingly, cleavage of some of the HAs by TMPRSS2 resulted in an aberrant cleavage profile as compared to cleavage of the same HA by trypsin. Typically, TMPRSS2-mediated cleavage resulted in HA_1_ and HA_2_ subunits with slightly faster migration on SDS-PAGE (Figure 5A, H2, H5, and H10), as well as multiple cleavage products (Figure 5A, H10 and WSN). It is unclear why the TMPRSS2-mediated HA cleavage profiles are different from those obtained with HAT and trypsin, but it is possible that TMPRSS2 is cleaving at multiple sites within HA or that it is cleaving a specific glycoform of these HAs, either of which may explain the relative differences in mobility.

The H5 and H7 HAs contain polybasic cleavage site sequences, so discerning whether cleavage observed in the presence of HAT or TMPRSS2 was due to furin-like proteases or the transfected protease would be impossible given the confines of these experiments; therefore, they were generally excluded from analysis. However, Figure 5A shows the gel images for the H5 and H5^VN^ HAs. While the level of cleavage observed when these HAs were co-expressed with HAT was indistinguishable from the level of cleavage observed in the absence of trypsin, when they were co-expressed with TMPRSS2, we observed an increase in the percent of total cleaved HA. As such, the cleavage efficiency of these HAs in the presence of TMPRSS2 was included in the quantitative analysis (Figure 5C). Importantly, for the H5 and H5^VN^ HAs, the percent cleavage observed in the absence of trypsin was subtracted from the percent cleavage observed in the presence of TMPRSS2, which is what is expressed in Figure 5C.

### The pH of HA-mediated membrane fusion varies between and within subtypes

**Figure 6:**
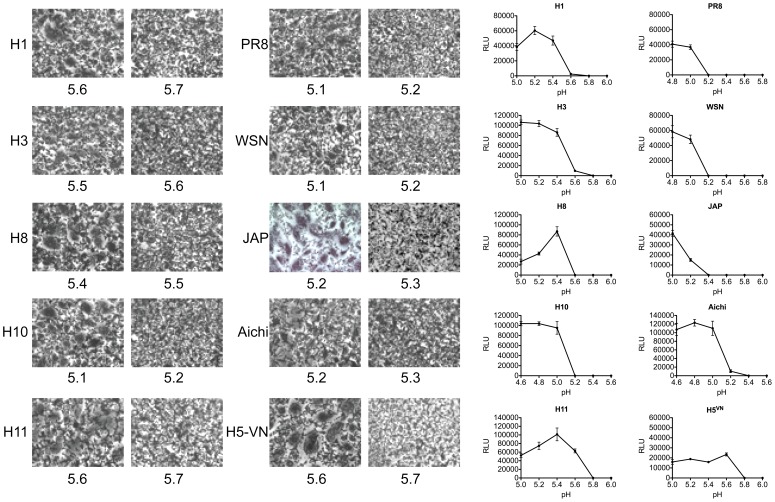
The pH of fusion for a select representative group of HA proteins. (**A**) Photomicrographs of syncytia formation assay. BHK cells were transfected with 1.0 µg HA plasmid. At 16 hrs post transfection, HA-expressing BHK cells were treated with TPCK-trypsin (5 µg/ml), followed by treatment with pH-adjusted PBS in 0.1 pH unit increments, neutralized, and incubated at 37°C for 2 hr. Cells were then washed with PBS, fixed, stained, and imaged. Photomicrographs corresponding to the last pH at which syncytia were observed and 0.1-pH unit higher are shown. (**B**) Luciferase reporter gene assay for fusion. Vero cells were co-transfected with 1.0 µg HA plasmid and 1.0 µg T7-Luciferase plasmid. At 16 hrs post transfection, HA-expressing Vero cells were treated with TPCK-trypsin (5 µg/ml), followed by treatment with *C. soybean* trypsin inhibitor (20 µg/ml). HA-expressing Vero cells were overlaid with BSR-T7/5 target cells that constitutively express T7 RNA polymerase. The two cell populations were incubated for 1 hr, at which time cell monolayers were pulsed with pH-adjusted PBS in 0.2 pH unit increments at the indicated pH for 5 min to trigger fusion, and then were neutralized. Cells were incubated for 6 hrs at 37°C to allow for cell-to-cell fusion to occur, which would mediate transfer of the T7-luciferase plasmid and expression of firefly luciferase. Luminescence was measured as an indicator of membrane fusion in cell lysates. The graphs show the mean background-adjusted relative luminescence (± standard deviation) as a function of pH obtained from three independent experiments.

**Figure 7:**
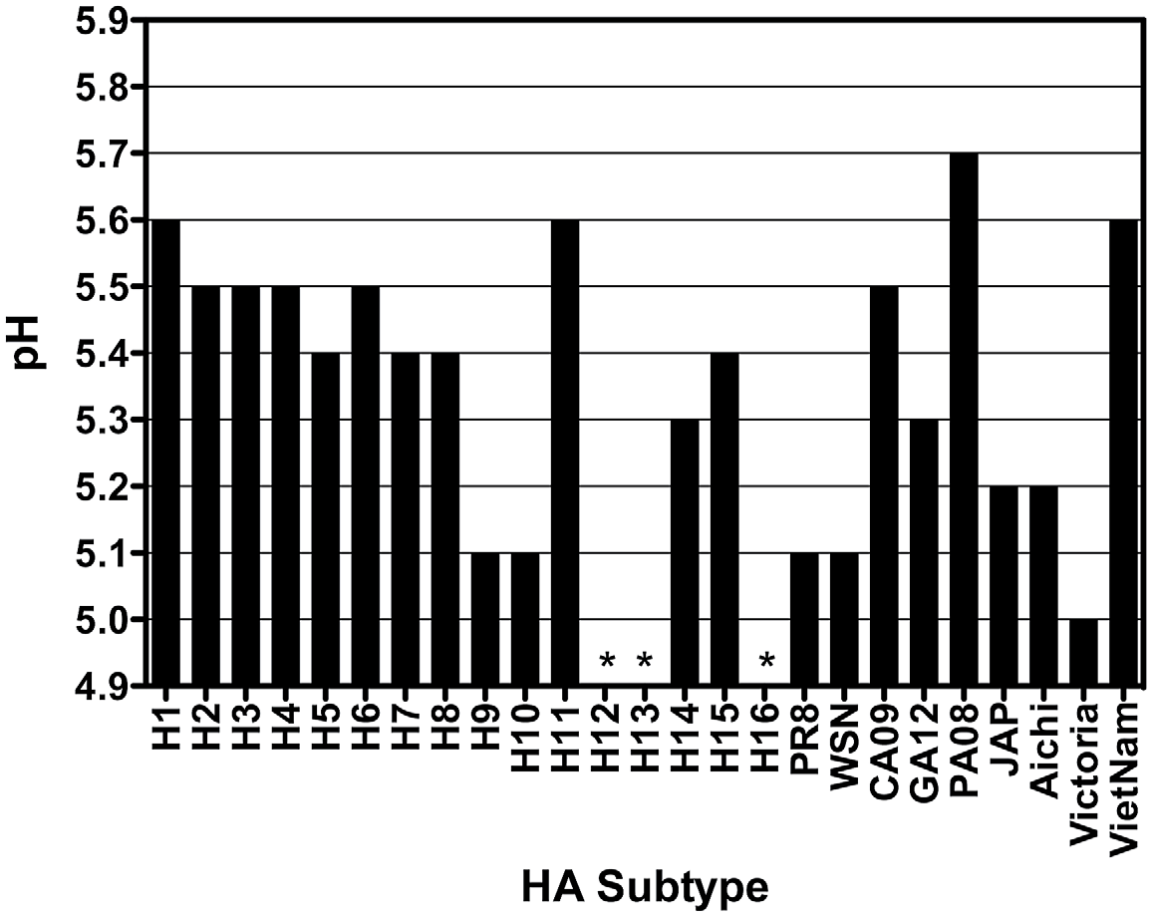
Summary of the pH of fusion obtained from the syncytium and luciferase assays for all subtypes examined. (*) indicates no fusion was detected.

The pH at which each HA protein mediated cell-to-cell fusion was measured by syncytia formation and luciferase reporter gene assays, as described in Material and Methods. Syncytia formation of proteolytically activated HA-expressing BHK cells was assessed 16 hrs post-transfection by incubation with low pH buffer in 0.1 pH unit increments, and the pH of fusion was defined as the highest pH value at which syncytia were observed (Figure 6A and Table 2). The pH of fusion was also determined using a quantitative luciferase-based cell-to-cell fusion assay, in which fusion is quantified based on the transfer and subsequent expression of a plasmid encoding firefly luciferase under the control of the T7 bacteriophage promoter from Vero cells to BSR-T7/5 target cells. Relative luminescence at each pH value was determined by subtracting the luminescence detected from Vero cells transfected with the T7-luciferase plasmid only, and the pH of fusion was defined as the highest pH value for which mock-corrected luminescence was detected. A representative set of data for the syncytia formation and luciferase fusion assays of trypsin-cleaved HAs is shown in Figure 6A and B. The data from both assays is shown graphically in Figure 7 for all HAs (also summarized in Table 2). The pH at which these HAs mediated cell-to-cell fusion varied within a range of 5.0–5.7, and there was consistent agreement between the two fusion assays for each HA.

**Table 2:** Summary of proteolytic cleavage and fusion data for all HAs examined.

	(−) Neuraminidaseβ		(+) Neuraminidaseβ				pH of Fusion^a^	
HA Subtype	(−) Trypsin	(+) Trypsin	(−) Trypsin	(+) Trypsin	(+) HATβ	(+) TMPRSS2β	Syncytia	Luciferase
H1	-	18	-	36	-	65	5.6	5.6
H2	-	34	-	41	10	59	5.5	5.4
H3	-	21	-	28	-	7	5.5	5.6
H4	-	16	7	34	4	77	5.5	5.4
H5	33	38	43	53	-	53	5.4	5.4
H6	1	23	2	69	2	69	5.5	5.4
H7	7	18	16	22			5.4	5.4
H8	-	41	-	40	-	-	5.4	5.4
H9	-	44	3	56	19	91	5.1	5.0
H10	-	30	-	61	-	75	5.1	5.0
H11	15	29	10	40	30	45	5.6	5.6
H12	-	4	-	6	36	-	ND	ND
H13	-	-	-	-	-	32	ND	ND
H14	-	16	-	61	1	60	5.3	5.2
H15	-	43	-	55	*	*	5.4	5.4
H16	-	-	-	-	-	22	ND	ND
H1^PR8^	-	27	-	44	7	75	5.1	5.0
H1^WSN^	-	53	29	67	6	59	5.1	5.0
H1^CA09^	-	19	-	20	-	41	5.5	5.6
H1^GA12^	/	/	/	/	/	/	5.3	5.4
H1^PA08^	/	/	/	/	/	/	5.7	5.8
H2^JAP^	-	26	-	33	11	24	5.2	5.2
H3^Aichi^	-	51	-	38	14	76	5.2	5.2
H3^Victoria^	/	/	/	/	/	/	5.0	5.0
H5^VN^	43	52	67	84	ND	44	5.6	5.6

ND - None Detected.

/ = Not Determined.

a - The disparity in the pH of fusion is likely due to the fact that the luciferase assay was done in 0.2 pH unit increments and the syncytia assay was done in 0.1 pH unit increments.

β - Values shown are percent cleavage of total HA expressed in transfected cells.

* Not determined due to poor antibody reactivity.

Consistent with the lack of trypsin-mediated cleavage of the H13 and H16 HAs, none of those HAs examined displayed any detectable cell-to-cell fusion, as measured by either the syncytium formation or luciferase fusion assays (Figure 7). In addition, fusion was not detected for the H12 HA, for which the level of trypsin-mediated cleavage observed was approximately 4% (Figure 7). It is possible that this low level of cleavage is not biologically significant enough to mediate fusion. Notably, we also attempted to assess the pH of fusion for the H12 HA in the presence of HAT rather than trypsin, but we were unable to detect fusion using the luciferase-based cell-to-cell fusion assay (data not shown). As shown above, the H11 HA was cleaved in the absence of trypsin; however, we were not able to detect cell-to-cell fusion mediated by the H11 HA in the absence of trypsin (data not shown).

### HA-mediated fusion of TMPRSS2-cleaved HAs

**Figure 8:**
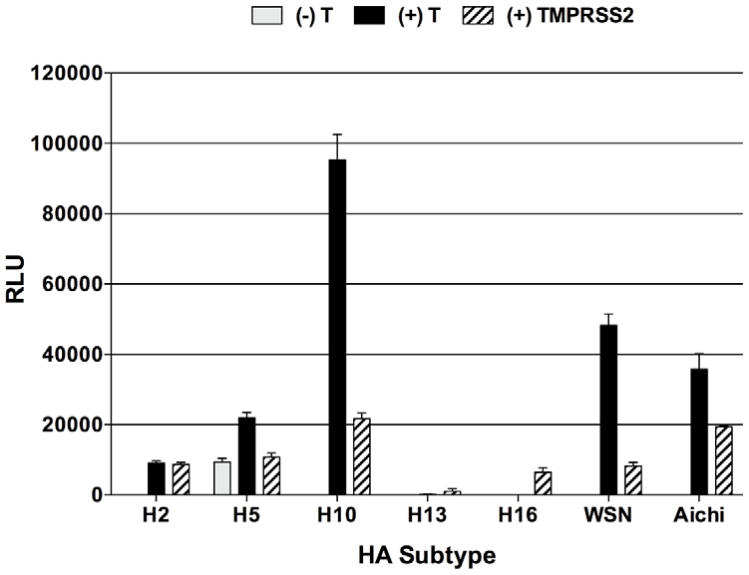
HA-mediated fusion in the absence of trypsin, the presence of trypsin, or the presence of TMPRSS2, as measured using the luciferase reporter gene fusion assay. Vero cells were transfected with 1.0 µg HA plasmid, 1.0 µg T7 luciferase plasmid, and, where indicated, 0.25 µg TMPRSS2 plasmid. At 16 hours post transfection, HA-expressing Vero cells were either left untreated or treated with TPCK-trypsin (5 µg/ml), and overlaid with BSR-T7/5 target cells. Cells were then treated with PBS or PBS that had been pH adjusted to 5.0 with citric acid, neutralized, and incubated at 37°C for 6 hr to allow for cell-to-cell fusion to occur. Cell populations were then harvested and the luciferase activity resulting from the fused cell populations was quantified and is expressed as the mean ± standard deviation of relative luminescence units (RLU).

Given the aberrant cleavage patterns and altered migration of the HA subunits liberated upon TMPRSS2 cleavage, we wanted to determine whether the TMPRSS2-cleaved HAs were in fact fusogenic. In addition to examining fusion for the TMPRSS2-cleaved H2, H5, H10, and WSN HAs mentioned above, we also examined whether the H13 and H16 HAs were capable of mediating fusion. To examine fusion, we utilized the luciferase-based cell-to-cell fusion assay described above and in Materials and Methods. The data in Figure 8 show that, although these TMPRSS2-cleaved HAs have aberrant cleavage profiles compared to cleavage by trypsin, they are still capable of mediating fusion. Additionally, the TMPRSS2-cleaved H13 and H16 HAs are also capable of mediating fusion, albeit at a very low level (Figure 8).

## Discussion

IAV HA membrane fusion properties, in conjunction with the characteristics for protease activation of HA, a requirement for fusion, are critical factors that are likely involved in the ecology and transmission of IAVs in nature. Both properties have the potential to influence virus stability, as viruses with cleaved HAs can be prematurely triggered to undergo conformational changes, and thus inactivated, by environmental conditions prior to encountering permissive hosts or sites of infection. The environmental milieu encountered by IAVs in nature potentially exerts relatively varied selective pressures that may drive these viruses to evolve HAs to have properties that increase or decrease stability; however, any modulation of stability needs to be counterbalanced by the requirement for HA to be triggered by endosomal pH to undergo the irreversible conformational changes that initiate membrane fusion during virus entry. Of the 16 HA subtypes that circulate in aquatic birds, and thus have the potential to infect mammals, there is analysis of cleavage-activation and membrane fusion characteristics for only a very limited number of subtypes. In the present study, we examined a panel of HA proteins that were derived from the WHO reference strains representative of the 16 subtypes, as well as HA proteins derived from human viruses that are representative of subtypes that have infected humans over the last century, for their susceptibility to cleavage by trypsin as well as the human serine proteases, HAT and TMPRSS2. Additionally, we examined the pH at which each HA mediated membrane fusion. Our data have shown that, while nearly all HAs were cleaved by trypsin, HAT, and TMPRSS2, the efficiency of cleavage varied considerably from subtype to subtype, and some HAs were resistant to cleavage by certain proteases (Figures 2 and 5; Table 2). Further, we found that the pH of fusion varied by 0.7 pH units among the HA subtypes examined, as well as among HAs derived from viruses isolated from different hosts (Figures 6 and 7; Table 2). Overall, these data have implications for the stability, replication, and transmissibility of IAVs in various environmental conditions and host species.

Examination of the cleavage site sequences for all of the HAs utilized in this study did not reveal any overt rationale for the variations in cleavage efficiency for most of the HAs (Table 1). Generally, cleavage by trypsin is less affected by residues in the P_3_ and P_4_ positions, with the notable exceptions of Pro_3_ and Gly_4_, both of which reduce the efficiency of cleavage [52]. Similarly, trypsin shows little preference at the P_2_ position; however, charged residues in the P_2_ position significantly reduce cleavage efficiency [52]. This may be a contributing factor to the substantially low or undetectable cleavage observed for the H12 and H16 HAs, which contain Asp_2_ and Glu_2_, respectively.

To our knowledge, the optimal substrate for TMPRSS2 has not been characterized. Studies examining the activity of TMPRSS2 have typically utilized a broad trypsin substrate containing a Gly_3_Gly_2_Arg_1_ cleavage site, which only moderately resembles the cleavage site found in the H10 HA (Table 1). Most of the HAs examined in this study were cleaved efficiently by TMPRSS2, indicating that variations to this broadly utilized substrate are well tolerated. Of the HAs that were most efficiently cleaved by TMPRSS2, the only conserved cleavage site features consisted of having a small hydrophobic amino acid in the P_4_ position and a Ser/Thr in the P_2_ position, but these features are not absolute determinants for cleavage, as HAs containing other residues at these positions are also cleaved with varying efficiency by TMPRSS2. The optimal substrate shown to be hydrolyzed by HAT is Arg_4_Gln_3_Asp_2_Arg_1_ [53]. Of the HAs examined, the H12 HA cleavage site sequence shows the highest degree of similarity with Ala_4_Gln_3_Asp_2_Arg_1_, owing to the relatively robust cleavage of the H12 HA by HAT (Figure 5A and 5B).

The H13 and H16 HAs did not show any detectable cleavage by trypsin, despite the fact that they were expressed on the cell surface and available to exogenous trypsin. A recent study, in which the crystal structure of an H16 HA in its uncleaved HA_0_ form was solved, provides a potential explanation for the general lack of trypsin-mediated cleavage [54]. Comparison of the structure of the cleavage loop of H16 to the available H1 and H3 structures shows that the cleavage loop consists of a short α-helix that “hides” the cleavage site within the cavity found within the trimer interior that contains several ionizable residues believed to be important in triggering fusion. Unlike the H16 HA, the cleavage loop of the H3 HA extends away from the trimer and the cleavage site is situated far enough from the cavity that no contacts are formed until post-cleavage when the newly generated N-terminus of HA_2_ is relocated to the trimer interior. A similar structure is found with the H1 HA, except that the cleavage site abuts the HA, but remains accessible to protease [54]. Consistent with the observed lack of HA cleavage by trypsin, neither the H13 nor the H16 mediated fusion in the presence of trypsin, as assessed by our fusion assays. This phenotype provides a likely explanation for why H13 and H16 viruses were not efficiently propagated or able to form plaques in MDCK cells in the presence of trypsin; however, both viruses replicated to high titers in embryonated chicken eggs [55]. A recent study showed that H13 and H16-containing recombinant viruses were only recoverable when transfected 293T cells were injected into embryonated chicken eggs for propagation, indicating that a protease present in embryonated chicken eggs was necessary for cleavage and replication of H13 and H16 recombinant viruses [55]. While the H13 and H16 HAs were not cleaved by trypsin or HAT, they were both cleaved with similar efficiency by TMPRSS2 (32% and 22%, respectively), which was sufficient to provide a very modest level of fusion (Figure 8). Discriminate utilization of activating proteases may play a role in the ecology and host range of influenza viruses in nature. It is interesting that H13 and H16 viruses have not been isolated from as many avian species as other HA subtype viruses, but rather appear to be generally restricted to the order *Charadriiformes*.

The H12 HA was cleaved by trypsin at very low levels (approx. 4%); however, this level of cleavage was not sufficient to mediate cell-to-cell fusion, as assessed by our assays. Unlike all other HAs examined, the H12 HA was most efficiently cleaved by HAT, but even for the HAT-cleaved H12 HA we were unable to demonstrate fusion activity. In a recent study, a recombinant virus expressing the H12 HA examined in this study was found to replicate poorly in MDCK cells (titers of 10^4.4^), whilst replication in eggs was fairly robust (10^7^) [55]. Embryonated chicken eggs are known to express a Factor Xa-like serine protease, and the relative difference in titers obtained for the H12 viruses may reflect the activities of available proteases. In addition to the recognition of HA as a substrate for activating proteases, there is also the requirement for the activating protease to generate specific cleavage products containing fusion-active sequences at the HA_2_ N-terminus. The HA_2_ N-terminal fusion peptide domain that results from cleavage is the most conserved region in HA, with the sequence GLFGAIAGFIE being virtually invariant in natural isolates. HA digestion by proteases such as thermolysin, which cleaves between the G and L at position 2 of authentic HA_2_, render the HA inactive for fusion and results in non-infectious virus [23], [33], [56], [57]. It is possible, particularly for the H12 HA, that cleavage with the HAT protease does not generate an authentic HA_2_ N-terminus that is capable of mediating fusion.

During this study, we observed that trypsin-mediated cleavage was enhanced when HA and NA proteins were co-expressed. We expected to see greater levels of cleavage of the WSN HA when co-expressed with the WSN NA in the absence of trypsin due to its plasminogen/plasmin binding activity [47]. The majority of the NAs utilized in this study do not appear to stimulate HA cleavage in the absence of trypsin, with the exception of the H4, H6, and H9 HAs, and even these levels of cleavage are limited to less than 7% of total HA. The cognate NAs for the H4, H6, and H9 HAs do not contain the carboxy-terminal K in conjunction with the absence of the glycosylation site at position 146 (N2 numbering) that was shown to be required by the WSN NA for its plasminogen binding activity [48]. As such, it is unclear why co-expression of the H4, H6, and H9 NAs stimulate cleavage in the absence of trypsin, but nevertheless may be due to an endogenous serum protease. However, the presence of NA had a considerable effect on cleavage of HA in the presence of trypsin (Figure 2A and 2D, Table 2). While we do not have a definitive explanation for the increase in HA cleavage, we did observe that when HA and NA proteins are co-expressed in transfected cells, the total amount of HA on the surface of the cell is greater, providing more of the total cellular HA for cleavage upon addition of exogenous trypsin (Figure 4). We are currently assessing the impact of cognate and non-cognate NAs on surface expression and cleavage efficiency to determine whether this is another mechanism by which HA and NA functions balance one another in nature [58]–[62].

The HAs examined in this study all mediated membrane fusion between pH 5.0 and 5.7 (Figure 7, Table 2). Interestingly, we observed that most of the HAs derived from human isolates mediated membrane fusion 0.1–0.5 pH units lower than an HA of the same subtype that was derived from an avian species, with most differences being ≥0.3 pH units lower. The exceptions to the aforementioned disparity between the pH of fusion for avian-origin and mammalian-origin HAs were the HAs derived from A/VietNam/1203/2004 (H5N1), which mediated membrane fusion 0.2 pH units higher than the HA derived from A/tern/South Africa/1961 (H5N3), and A/Pennsylvania/08/2008 (H1N1), which mediated membrane fusion 0.1 pH units higher than A/duck/Alberta/35/1976 (H1N1). The H5N1 viruses have yet to acquire the ability to transmit efficiently between humans, as such, increasing acid stability of the HA may represent a potential for adaptation. In fact, a recent study showed that passage of a recombinant H5N1 virus in ferrets led to the acquisition of mutations, one of which increased the stability of HA, that increased the efficiency of droplet transmission between ferrets [3]. The higher pH of fusion observed for the A/Pennsylvania/08/2008 (H1N1) HA was particularly interesting. It was the only human HA examined that mediated membrane fusion higher than the avian HAs examined in this study. H1N1 viruses were reintroduced into the human population in 1977 and have continued to co-circulate with seasonal H3N2 viruses. However, it is interesting to note that among the circulating seasonal influenza viruses, the H1N1 viruses rarely predominated the H3N2 influenza viruses, and generally only did so following unusually mild H3N2 epidemics and were characterized by lower mortality rates [63]–[65], which is suggestive of greater fitness of H3N2 viruses than H1N1 viruses in the human population. It is possible that the acid stability of the HA protein is a contributing factor in the dynamics of seasonal epidemics of H1N1 and H3N2 viruses.

Although the A/California/04/2009 (H1N1) HA mediated membrane fusion at pH 5.5, only 0.1 pH unit lower than the HA from A/duck/Alberta/35/1976 (H1N1), the HA from a more recent H1N1 virus that shares >99% identity with the A/California/04/2009 (H1N1) HA mediated membrane fusion at pH 5.3. The A/California/04/2009 (H1N1) strain was a very early isolate from the 2009 pandemic and the higher pH of fusion may be indicative of its prior adaptation to the porcine host, whereas the A/Georgia/F32551/2012 (H1N1) presumably has been circulating in the human population and the lower pH of fusion may represent an adaptation to the human host.

Of the avian-origin HAs examined, the H9 and H10 HAs had greater acid stability than the other subtypes. Of these two subtypes, viruses having an H9 HA may be of greater significance, as H9N2 viruses are widespread in poultry in Asia and there have been reported cases of human infection with H9N2 IAVs [66]–[68]. On one hand, our data may indicate that H9 and H10-containing viruses have a smaller evolutionary barrier to overcome if transmitted to humans; however, we caution against this strict interpretation, as our study is limited to one isolate for most subtypes, and it would not be at all surprising to find a degree of variability in the pH of membrane fusion as more isolates are examined in the future. For example, it has been shown that the pH of fusion of pathogenic H7 subtypes from chicken isolates can vary by as much as 0.6 pH units [69], and a comparison of H7N3 viruses from turkeys and ducks showed that the viruses isolated from turkeys mediated membrane fusion below pH 6, whilst the viruses isolated from ducks still retained fusion activity at pH 6 [70].

In support of the concept that pH may play a role in adaptation or pathogenicity, a recent study examined the infectivity and immunogenicity of two NS1-deficient H5N1 (A/Viet Nam/1203/2004) viruses having either the wild-type HA or an HA encoding the K58I mutation in the HA_2_ subunit [71]. Previous studies on the H3, H7, and H5 HAs have shown that the HA_2_ K58I mutation decreases the pH at which the HA mediates viral membrane fusion by 0.7 pH units (H3 and H7) or 0.6 pH units (H5) [72], [73]. When the infectivity of the NS1-deficient viruses was examined after exposure to acidic buffer or heat, the virus having the K58I substitution retained infectivity after exposure to lower pH and higher temperatures. When virus replication in the mouse upper respiratory tract was examined, the virus having the K58I mutation was present in nasal tissue homogenates at higher titers than the virus with a wild-type HA, and also yielded higher titers of neutralizing antibodies; however, both viruses were capable of protecting mice from lethal challenge [71]. Interestingly, the virus expressing the acid-stable H5-K58I HA replicated less well in mammalian cell culture than the virus expressing the wild-type HA. It was shown previously that IAVs that have been passaged in embryonated chicken eggs rapidly selected for HA mutants that mediated membrane fusion at higher pH when passaged in mammalian cells [74]. If acid-stability of HA is a phenotype that mediates more robust viral replication in mammalian species, it is possible that the aforementioned selection of acid labile HA proteins represent an adaptation to the cell culture system and does not necessarily represent a host-specific adaptation. Perhaps the most reliable means to examining species-specific adaptation as it relates to fusion is through surveillance and passage experiments *in vivo*, as there are several environmental factors that are not recapitulated in most cell culture systems that may drive the evolution of the HA more than most other IAV proteins given its exposure on the surface of the cell/virion. Examples of these conditions include the acidification of the nasal passageway in response to irritation or inflammation [75], [76], the temperature, salinity, and pH of waterways, and the pH of the intestinal tract where many avian viruses replicate. Along these lines, analysis of H5N1 IAV isolates from chickens that were considered either highly pathogenic or moderately pathogenic viruses showed that the pathogenicity was modulated by the acid stability of the HA protein. Specifically, the highly pathogenic IAV encoded for an HA that mediated membrane fusion at a higher pH than the HA from the moderately pathogenic IAV [77], indicating that acid lability of HA may be a contributing factor in determining pathogenesis in avian systems. In a separate study, using recombinant H5N1 viruses that were engineered to contain mutations previously found to alter the pH of fusion, it was shown that a relatively high pH of fusion was essential for infectivity, shedding, and transmission in ducks [78].

Additionally, a more recent study has shown that experimental acquisition of four mutations in the H5 HA from A/VietNam/1203/2004 (H5N1), one of which decreases the pH at which the HA mediates membrane fusion results in more efficient respiratory droplet transmission between ferrets [3], [4]. Of the four mutations introduced or selected for in the H5 HA, two were involved in increasing the ability of the H5 HA to bind to Siaα2,6Gal (N224K and Q226L), one resulted in the loss of a glycosylation site on the globular head of the HA (N158D), and one, T318I, was responsible for the increased stability observed for the H5 HA. While the presence of all four mutations resulted in the most efficient respiratory droplet transmission between ferrets, a virus having three mutations (N158D/N224K/Q226L) in the H5 HA was sufficient to mediate a modest level of transmission, and a virus having only the T318I mutation in HA that increases stability was unable to mediate respiratory droplet transmission between ferrets [3], indicating that transmissibility is a multifactorial trait that is influenced by a variety of phenotypes.

Although the avian viruses examined in our study were not direct progenitors of the human viruses, the collective data on avian and human HAs within a given subtype show an overall trend to suggest that the acid stability of the HA may play a role in adaptation to humans. However, the data for A/Pennsylvania/08/2008 (H1N1) HA indicate that a low pH phenotype is not a property of all circulating human viruses, and it is yet to be determined whether any adaptive effects of HA stability will be subtype or strain dependent, or if the contributions of other factors in a given genetic backbone may have superseding influence in the competitive environment. Furthermore, it may prove that once viruses are adapted, other selection criteria such as immune pressure, or drug-resistance to compounds such as α-adamantanes, exert overriding effects on the fusion pH phenotype. Additionally, it is well established that there are many mutations that can alter the acid stability of HA and that they occur throughout the length of the protein. This increases the likelihood that the selection of a specific mutation within HA in response to a particular host pressure may also have the ability to influence another function of HA. Nevertheless, it will be interesting to continue to monitor and compare the fusion pH of avian and human viruses in order to ascertain whether this property plays a role in adaptation and/or viral fitness in different species.

Distinct selective pressures on IAVs in mammalian versus avian systems govern differential replication of IAV isolates in various host species. Known adaptation traits for IAVs include modifications to proteins that alter the replicative efficiency of the RNA synthetic machinery, alterations to proteins that affect modulation of the host immune system, and modifications to the HA that affect cleavage-activation, receptor binding capability and specificity, as well as membrane fusion. The pH of fusion and the susceptibility of HA to host cell proteases may have broad implications for the stability and transmissibility of IAVs in nature, as well as the ability of viruses to infect and replicate in various host cell tissues (e.g. respiratory tract of humans, intestinal tracts of wild birds) and in evading the effects of anti-viral compounds [73]. The studies reported here emphasize that protease activation and membrane fusion properties of influenza A viruses may vary substantially, and that these may reflect varying ecological niches to which the different subtypes and strains have adapted. Overall, HA cleavability and acid stability has been shown to vary widely among HA subtype and host species, and is suggested to impact cross-species transmission, pathogenesis, and the emergence of novel influenza virus strains in humans.

## Materials and Methods

### Cells and viruses

BHK-21 cells (ATCC CCL-10) were used for transfection of plasmids to analyze fusion by syncytia formation and to analyze cleavage activation by select proteases. Vero cells (ATCC CCL-81) were used for transfection of plasmids to analyze fusion by the quantitative cell-cell fusion assay, described below. BSR-T7/5 cells (originally obtained from Karl-Klaus Conzelmann) were used as target cells in the cell-cell fusion assay [79].

WHO reference strains of *influenza A virus* were obtained from Dr. Alan Hay at the MRC National Institute for Medical Research (Mill Hill, London). The A/Victoria/3/1975 (H3N2) virus was obtained from BEI Resources.

### Plasmids

To simplify cloning of the HA and NA genes into pCAGGS [80], we engineered the pCAGGS vector to utilize ligation-independent cloning (LIC). The sequence between the EcoRI and BglII restriction sites of pCAGGS was replaced with a cassette encoding the chloramphenicol acetyl transferase (CAT) gene as well as the ccdB lethal gene that was derived from the Reading Frame C.1 (RFC.1) cassette in the Gateway Vector Conversion System (Invitrogen). From the RFC.1 template, a fragment corresponding to nucleotides 141–1584 was amplified by PCR using primers that encoded a 15-nucleotide sequence for LIC purposes and the SwaI recognition sequence. The fragment was cloned into pCAGGS between the EcoRI and BglII restriction sites. The resulting sequence between the EcoRI and BglII restriction sites is 5′ (GAATTC)_EcoRI_ CTGTACTTCCAATCC (ATTTAAAT)_SwaI_ -nucleotides 141–1584 of RFC.1- (ATTTAAAT)_SwaI_ GGAAGTGGATAACGG (AGATCT)_BglII_ 3′, with the EcoRI, SwaI, and BglII recognition sequences denoted in parentheses. The newly generated plasmid was named pCAGGS-LIC with a total size of 6.2 kb.

For cloning purposes, each HA and NA gene was amplified by RT-PCR from viral RNA obtained from the viruses listed in Table 1 using a (+) sense primer having the 5′ LIC-specific flanking sequence of 5′ TACTTCCAATCCATTTGCCACCATG , with the start codon underlined, and a (−) sense primer having the 5′ LIC-specific flanking sequence of 5′ TTATCCACTTCCATTTGTCA , with the stop codon underlined. In addition to the sequences shown, each primer had an additional 12–18 nucleotides of gene-specific sequence following either the start or stop codon. We utilized standard LIC procedures for generation of each HA and NA plasmid. Briefly, the pCAGGS-LIC vector was digested with SwaI and the 4.7 kb fragment was gel purified and utilized in a T4 DNA polymerase reaction with dGTP as the only nucleotide source. HA and NA genes were amplified by RT-PCR using the LIC primers described above, purified, and utilized in a T4 DNA polymerase reaction with dCTP as the only nucleotide source. The T4 DNA polymerase-treated vector and PCR product were incubated together on ice for 30 min to ligate the complementary 16 bp overhangs generated during T4 DNA polymerase reactions and transformed into competent cells. Clones were initially screened by restriction digestion with either EcoRI or BamHI and positive clones were further verified by sequence analysis.

Plasmids encoding human airway trypsin-like protease (HAT) or transmembrane protease, serine 2 (TMPRSS2) were kindly provided by Gary Whittaker (Cornell University, Ithaca, NY).

### Antibodies

The HA-specific antibodies utilized in this study are detailed below. From BEI Resources, we obtained the following: goat anti-H1 HA (A/duck/Alberta/35/1976_H1N1), goat anti-H2 HA (A/duck/Germany/1215/1973_H2N3), goat anti-H2 HA (A/Singapore/1/1957_H2N2), goat anti-H3 HA (A/duck/Ukraine/1/1963_H3N8), goat anti-H4 HA (A/duck/Czechosovakia/1956_H4N6), goat anti-H5 HA (A/tern/South Africa/1961_H5N3), goat anti-H7 HA (A/Canada/RV444/2004_H7N3), goat anti-H8 HA (A/turkey/Ontario/6118/1968_H8N4), goat anti-H10 HA (A/chicken/Germany/N/49_H10N7), goat anti-H11 HA (A/duck/England/1956_H11N6), goat anti-H1 HA (A/Puerto Rico/8/1934_H1N1), goat anti-H5 HA (A/Vietnam/1203/2004_H5N1). The rabbit anti-H12 (A/mallard/Sweden/81/2002_H12N5) and rabbit anti-H16 (A/black headed gull/Sweden/5/1999_H16N3) serum was kindly provided by Ron Fouchier (Erasmus Medical Center, Rotterdam, The Netherlands). For detection of the Aichi HA, we utilized rabbit anti-X-31 serum. The chicken anti-H15 HA antibody was obtained from Veterinary Laboratories Agency. The goat anti-H6 (A/shearwater/Australia/1/1972_H6N5), goat anti-H9 (A/turkey/Wisconsin/1/1966_H9N2), goat anti-H13 (A/gull/Maryland/704/1977_H13N6), and goat anti-H14 (A/duck/Astrakhan/263/1982_H14N5) were kindly provided by Alan Hay (MRC National Institute for Medical Research, Mill Hill, London).

### Fusion assays

HA-mediated membrane fusion was examined using two independent assays: a qualitative syncytia assay and a quantitative cell-cell fusion luciferase reporter assay. Syncytia assays were done as described previously [72]. Briefly, BHK-21 cells were transfected with 1.0 µg of HA plasmid using Lipofectamine (Invitrogen), according to the manufacturer's recommendation. Where noted, 0.25 µg HAT or 0.25 µg TMPRSS2 plasmids was included in the transfection. At 16–18 hrs post transfection, HA-expressing BHK cells were washed once with PBS and treated with 5 µg/ml TPCK-Trypsin (Sigma) at 37°C for 15 min. Trypsin-treated HA-expressing cells were subsequently exposed to 1.0 ml PBS that had been pH adjusted using 100 mM citric acid to the indicated pH and incubated at 37°C for 5 min. The pH-adjusted PBS was removed and HA-expressing cells were neutralized by the addition of complete growth medium and incubated at 37°C for 2 hr to allow syncytia to form. Cells were subsequently stained with the Hema3 Stat Pak according to the manufacturer's recommendation. Syncytia were visualized and photographed using a Zeiss Axio Observer inverted microscope with attached digital camera.

The quantitative luciferase cell-cell fusion assay was done as previously described [72]. Briefly, Vero cells were co-transfected with 1.0 µg of HA plasmid and 1.0 µg of a plasmid expressing firefly luciferase under control of the T7 bacteriophage promoter using Lipofectamine (Invitrogen), according to the manufacturer's recommendation. Where noted, 0.25 µg HAT or 0.25 µg TMPRSS2 plasmid was included in the transfection. At 16 hrs post transfection, HA-expressing Vero cells were washed once with PBS and treated with 5 µg/ml TPCK-Trypsin (Sigma), where indicated, at 37°C for 30 min. Trypsin-treated/untreated HA-expressing cells were subsequently overlaid with BSR-T7/5 target cells, which constitutively express the bacteriophage T7 RNA polymerase, and BSR-T7/5 cells were allowed to settle and adhere for 1 hr at 37°C. Following overlay with BSR-T7/5 target cells, cells were washed once with PBS and exposed to 1.0 ml PBS that had been pH adjusted using 100 mM citric acid to the indicated pH and incubated at 37°C for 5 min. The pH-adjusted PBS was removed and HA-expressing cells were neutralized by the addition of complete growth medium and incubated at 37°C for 6 hrs to allow for HA-expressing cells to fuse with BSR-T7/5 target cells in order to mediate transfer and expression of the T7-luciferase plasmid in BSR-T7/5 target cells. At 6 hrs post-neutralization, cells were washed once with PBS, lysed with 0.5 ml Reporter Lysis Buffer (Promega) and clarified of cell debris by centrifugation at 15,000× g at 4°C. From each clarified lysate, 150 µl was transferred to a 96-well plate (white polystyrene, Corning) and the luciferase activity resulting from the fused cell populations was quantified using a BioTek Synergy 2 Luminometer, using 50 µl of luciferase assay substrate (Promega) injected into each sample.

### Protease cleavage and immunoprecipitation

BHK, Vero, or DF-1 cells were transfected with 1.0 µg of HA plasmid using Lipofectamine (Invitrogen), according to the manufacturer's recommendation. Where noted, 0.1 µg NA, 0.5 µg NA, 1.0 µg NA, 0.25 µg HAT, or 0.25 µg TMPRSS2 plasmids was included in the transfection. At 16–18 hrs post transfection, HA-expressing BHK cells were washed twice with PBS and starved for methionine and cysteine by incubation in MEM (- Met-Cys) at 37°C for 30 min. Transfected cell proteins were subsequently pulse labeled by the addition of 25 µCi [^35^S]-methionine (Perkin Elmer) to the starvation medium and incubated at 37°C for 15 min, at which point cells were washed with PBS and chased with complete growth medium supplemented with 20× cold methionine at 37°C for 3 hr. After the chase period, cells were washed once with PBS and treated with either DMEM or DMEM supplemented with 5 µg/ml TPCK-Trypsin (Sigma) at 37°C for 15 min. Where noted, surface proteins were biotinylated with 2 mg EZ-Link Sulfo-NHS-SS-Biotin (Thermo Scientific) in PBS, pH 8.0 at ambient temperature for 30 min. Biotinylation reactions were quenched by washing cells twice with PBS, pH 8.0 supplemented with 50 mM glycine. Cells were then washed once with ice-cold PBS and lysed with 0.5 ml RIPA buffer [50 mM Tris-HCl, pH 7.4; 150 mM NaCl; 1 mM EDTA; 0.5% deoxycholate; 1% triton X-100; 0.1% SDS]. Cell lysates were centrifuged at 16,000× g for 10 min to pellet cell debris and cleared lysates were harvested.

Cell lysates were pre-cleared with 50 µl Protein-G Dynabeads (Invitrogen) at ambient temperature for 30 min. Protein-G Dynabeads were conjugated to HA-specific antibody in PBS+0.02% Tween-20 at ambient temperature for 30 min and unbound antibody was washed away from Protein-G dynabeads with PBS+0.02% Tween-20. Protein-G pre-cleared lysates were subsequently incubated with Protein-G/antibody complexes at ambient temperature for 1 hr. Antigen/antibody/Protein-G complexes were washed 3× with PBS, resuspended in 30 µl 1× SDS sample buffer, boiled for 5 min, and the bead eluate was harvested. For the H15 immunoprecipitations, after cell lysates were pre-cleared with 50 µl Protein-G Dynabeads, cleared lysates were incubated with the chicken anti-15 HA antibody for 1 hr with rocking. Antigen/Antibody complexes were subsequently incubated at ambient temperature for 1 hr with Protein-G Dynabeads that had been conjugated to a goat anti-chicken IgY antibody. Antigen/Antibody/Protein-G complexes were washed and processed as stated above.

When immunoprecipitating HA from cells whose surface proteins had been biotinylated, antigen/antibody/Protein-G complexes were washed 3× with PBS, resuspended in 50 µl 50 mM Tris-HCl+0.5% SDS, boiled for 5 min, and the bead eluate was harvested. Of the 50 µl eluate, 25 µl was subsequently immunoprecipitated with Streptavidin M-280 Dynabeads (Invitrogen) to pull down the cell-surface expressed HA and 25 µl served as the total HA sample. Briefly, 100 µl of Streptavidin M-280 Dynabeads were washed 3× with PBS+0.2% BSA and resuspended in 975 µl Streptavidin binding buffer [20 mM Tris-HCl, pH 8.0; 150 mM NaCl; 5 mM EDTA; 1% Triton X-100; 0.2% BSA], to which 25 µl of the eluate was added. The mixture was incubated at ambient temperature for 1 hr, at which time the streptavidin bound HA was washed 3× with PBS+0.2% BSA, resuspended with 35 µl 1× SDS sample buffer, boiled for 5 min, and the bead eluate was harvested. For gel analysis, the 25 µl total HA fraction was brought to a V_f_ of 35 µl with 3× SDS sample buffer and 20 µl of each sample was resolved on a 12% SDS-PAG.

SDS-PA gels were fixed with 50% Methanol/10% Acetic Acid for 30 min at ambient temperature and fixed gels were dried under vacuum for 1 hr at 80°C prior to being exposed to a phosphorimaging screen. Phosphor screens were scanned on a Typhoon Trio variable mode imager (GE Healthcare Life Sciences). The intensity of the bands corresponding to HA_0_, HA_1_, or HA_2_ was determined using the ImageQuant software, normalized to the methionine content of the appropriate subunit, and percent cleavage was determined using the equation (HA_2_/(HA_2_+HA_0_))×100%. For analysis of cell surface-expressed HA, the total level of HA in each lane was determined and the percent of surface expressed HA was determined by the following equation, (cell surface HA/total HA)×100%.
